# Long-Term Survival Advantage of Total Arterial Revascularization in Elderly Patients Following Coronary Artery Bypass Grafting

**DOI:** 10.1016/j.jacadv.2025.102226

**Published:** 2025-10-08

**Authors:** Justin Ren, Christopher M. Reid, Julian A. Smith, Colin Royse, Dion Stub, Wiliam Chan, David M. Kaye, Jason E. Bloom, Nilesh Srivastav, Andrea Bowyer, David H. Tian, Lavinia Tran, Jenni Williams-Spence, Doa El-Ansary, Alistair Royse

**Affiliations:** a Department of Surgery, University of Melbourne, Melbourne, Australia; b Department of Cardiothoracic Surgery, Royal Melbourne Hospital, Melbourne, Australia; c Population Health, Curtin University, Perth, Australia; d School of Public Health and Preventive Medicine, Monash University, Melbourne, Australia; e Department of Surgery, Monash University, Melbourne, Australia; f Department of Cardiothoracic Surgery, Monash Health, Melbourne, Australia; g Department of Anesthesia and Pain Management, The Royal Melbourne Hospital, Melbourne, Australia; h Outcomes research, University of Texas Health, Houston, USA; i Department of Cardiology, The Alfred Hospital, Melbourne, Australia; j Baker Heart and Diabetes Institute, Melbourne, Australia; k Department of Cardiology, Western Health, Melbourne, Australia; l Heart Vascular and Thoracic Institute, Cleveland Clinic Abu Dhabi, Abu Dhabi, United Arab Emirates; m Department of Anesthesia and Perioperative Medicine, Westmead Hospital, Sydney, Australia; n School of Biomedical and Health Sciences, RMIT, Melbourne, Australia; o Department of Surgery, Universiti Kebangsaan Malaysia, Kuala Lumpur, Malaysia

**Keywords:** coronary artery bypass grafting, elderly population, inverse probability treatment weighting, multiple arterial grafting, survival analysis, total arterial revascularization

## Abstract

**Background:**

Despite the evidence of clinical benefit, total arterial revascularization (TAR) remains underutilized in elderly patients undergoing coronary artery bypass grafting due to concerns about perceived surgical complexity and limited life expectancy.

**Objectives:**

The objective of the study was to evaluate long-term survival of TAR vs conventional non-TAR grafting strategies in elderly (≥70 years) and younger (<70 years) patients using a binational cardiac surgery registry.

**Methods:**

The study included patients who underwent primary isolated coronary artery bypass grafting with at least 2 grafts between 2001 and 2020. The endpoint was long-term all-cause mortality. Patients were stratified into 2 age groups, <70 years and ≥70 years. Within each cohort, survival outcomes were compared between those who received TAR, and those who received non-TAR involving at least 1 saphenous vein graft. Secondary analyses further divided the non-TAR group into patients receiving multiple arterial grafting or single arterial grafting. Baseline differences were adjusted using inverse probability treatment weighting, followed by Cox proportional hazard modeling.

**Results:**

Among 59,641 patients, TAR was associated with significantly improved survival compared to non-TAR in both elderly (HR: 0.87; 95% CI: 0.81-0.92; *P* < 0.001) and younger age groups (HR: 0.80; 95% CI: 0.73-0.88; *P* < 0.001). A clear hierarchy in survival was also demonstrated, with the highest survival observed in patients undergoing TAR, followed by non-TAR-multiple arterial grafting, and the lowest in those receiving non-TAR-single arterial grafting.

**Conclusions:**

TAR improves long-term survival in both elderly and younger patients. These findings challenge the assumption that limited life expectancy precludes arterial grafting and support broader implementation of TAR in appropriately selected older patients. Randomized clinical trials evaluating TAR are warranted to validate these observational findings.

Between 1950 and 2021, global life expectancy has increased by 22.7 years, from 49.0 to 71.7 years at birth,^1^ largely due to advances in public health measures and medical care. This demographic shift has led to a growing proportion of older adults, for whom coronary artery disease (CAD) poses a major health burden. Age is among the strongest nonmodifiable risk factors for CAD, contributing to CAD’s both rising incidence and clinical complexity.^2^ Elderly patients, compared to their younger counterparts, often present with diffuse, multivessel disease that often requires surgical revascularization with coronary artery bypass grafting (CABG). As a result, the aging population has intensified the imperative to evaluate and optimize surgical coronary revascularization strategies, particularly with respect to long-term patient outcomes.

The long-term benefits of total arterial CABG have been well documented relative to conventional strategies that employ a single left internal mammary artery to the left anterior descending artery, supplemented by saphenous vein grafts (SVGs).^3^, ^4^, ^5^ These advantages are supported by angiographic evidence demonstrating superior durability and resistance to atherosclerosis of arterial conduits relative to vein grafts.^6^^,^^7^ Nevertheless, the global adoption of total arterial revascularization (TAR) remains limited, particularly among elderly patients,^8^ in part related to concerns of perceived technical complexity or longer operative duration associated with arterial revascularization.^9^, ^10^, ^11^ These factors are further exacerbated by the higher prevalence of preoperative comorbidities and postoperative complications in elderly populations.

To date, few studies have specifically evaluated TAR in elderly patients. This is potentially due to the prevailing belief that individuals with limited life expectancy may not live long enough to fully realize the long-term benefits of arterial grafts, which are often considered to provide delayed rather than immediate survival advantages. In addition, the global underutilization of TAR has resulted in limited availability of analyzable patient data in most registries, further contributing to this critical evidence gap. This nationwide study primarily aims to evaluate long-term survival outcomes in both elderly and younger patients undergoing primary isolated TAR, compared with conventional CABG procedures with the use of SVG (non-TAR). The secondary aim is to further explore survival within the non-TAR cohort, stratified by multiple arterial grafting (non-TAR-MAG) vs single arterial grafting (non-TAR-SAG).

## Methods

A prospectively collected cardiac surgery registry, established by the Australian and New Zealand Society of Cardiac and Thoracic Surgeons (ANZSCTS), was used in this complete-case retrospective cohort study. Local project-specific approval was obtained from the Melbourne Health Institutional Human Research Ethics Committee (HREC 2011.164) with a waiver of individual patient consent due to the study’s retrospective design and the use of nonidentifiable, encrypted data. The Registry serves as a clinical quality assurance and research resource, with data contributed by 63 participating hospitals under an opt-out consent model. Detailed preoperative, intraoperative, and postoperative variables were collected via case report forms, which were collaboratively completed by surgical and clinical staff. All data underwent rigorous validation before entry into a secure web-based platform. Data sets were accessed through the Monash Secure eResearch Platform, a secure and auditable computing environment that prevents external data export and protects against reidentification. Only aggregated results were exported following approval by the ANZSCTS research program managers.

### Study populations

All adult patients who underwent primary isolated CABG with at least 2 grafts between 2001 and 2020 were identified from the ANZSCTS Cardiac Surgery Registry. Patients were excluded if they had a history of prior cardiac surgery, underwent concomitant cardiac procedures, or received only a single graft or no arterial grafts.

### Outcomes

The endpoint of this study was long-term all-cause mortality, measured from the date of index operation to the event date, obtained via the Australian Institute of Health and Welfare’s National Death Index.

### Statistical analyses

All statistical analyses were conducted using R (version 4.0.5; R Foundation for Statistical Computing). Baseline characteristics were summarized as mean (SD) or median (IQR) for continuous variables, depending on the normality distribution, and as counts and percentages for categorical variables. A 2-tailed *P* value of <0.05 was considered statistically significant. All analyses were performed on a per-anastomosis basis.

In the primary analyses, patients were categorized into 2 groups based on the graft strategy. The TAR group comprised procedures in which only arterial conduits were used, with complete avoidance of SVGs. The non-TAR group included patients who received at least 1 SVG during the procedure. For secondary analyses, the non-TAR group was further stratified based on the number of arterial grafts used: patients receiving ≥2 arterial grafts were classified as non-TAR-MAG, whereas those receiving <2 arterial grafts were categorized as non-TAR-SAG.

To adjust for baseline differences between treatment groups, propensity scores were estimated using a generalized linear model based on a comprehensive set of preoperative and perioperative covariates. These included demographic characteristics (age, sex, and smoking history), cardiovascular risk factors (hypertension, hypercholesterolemia, and diabetes mellitus), comorbid conditions (dialysis dependence, cerebrovascular disease, peripheral vascular disease, and chronic respiratory disease), and clinical history (prior myocardial infarction, infective endocarditis, arrhythmia, and cardiogenic shock). Additional variables reflecting cardiac function and disease severity were included, such as NYHA and Canadian Cardiovascular Society classifications, left ventricular ejection fraction, creatinine level, body mass index, presence of left main CAD, and the number of diseased coronary territories. Operative characteristics such as the number of grafts, use of cardiopulmonary bypass (on-pump surgery), operative urgency, and year of surgery were also incorporated to ensure robust adjustment.

Inverse probability of treatment weighting (IPTW) with marginal stabilization was applied using the estimated propensity scores to create a weighted population with balanced baseline covariates between the comparative groups. Covariate balance was assessed using standardized mean differences (SMDs), with a threshold of <0.1 considered indicative of adequate adjustment.^12^

The primary analysis focused on comparing the long-term survival differences between TAR and non-TAR procedures within 2 prespecified age groups—elderly patients (aged ≥70 years) and younger patients (<70 years)—to assess whether the treatment effect varied by age subgroup. Separate IPTW-weighted Cox proportional hazards models were constructed for each age group to estimate adjusted HRs and 95% CIs. The proportional hazards assumption was tested using weighted Schoenfeld residuals. Adjusted Kaplan-Meier survival curves were generated using the IPTW-weighted models to visualize differences in mortality over time across treatment strategies within each age group.

The secondary analysis evaluated long-term survival outcomes across three distinct graft strategies, including TAR, non-TAR-MAG, and non-TAR-SAG, within both elderly and younger populations. Additional IPTW-weighted Cox proportional hazards models, adjusted for the same set of baseline and operative variables, were used to assess whether the use of SVGs or the number of arterial conduits influenced clinical outcomes.

### Sensitivity analyses

To assess the robustness of the treatment effect estimates obtained through IPTW, a sensitivity analysis was conducted using propensity score matching (PSM) as an alternative adjustment method. A one-to-one nearest-neighbor matching algorithm was implemented without replacement, applying a caliper of 0.2 SDs of the logit-transformed propensity score to limit matches to sufficiently similar individuals. All baseline and operative covariates used in the primary analysis were included in propensity-score calculation during PSM to optimize covariate balance. A robust sandwich variance estimator was employed to account for clustering within matched pairs. Matched Kaplan-Meier survival curves were subsequently generated to visualize long-term mortality differences between TAR vs non-TAR.

## Results

**Figure 1:**
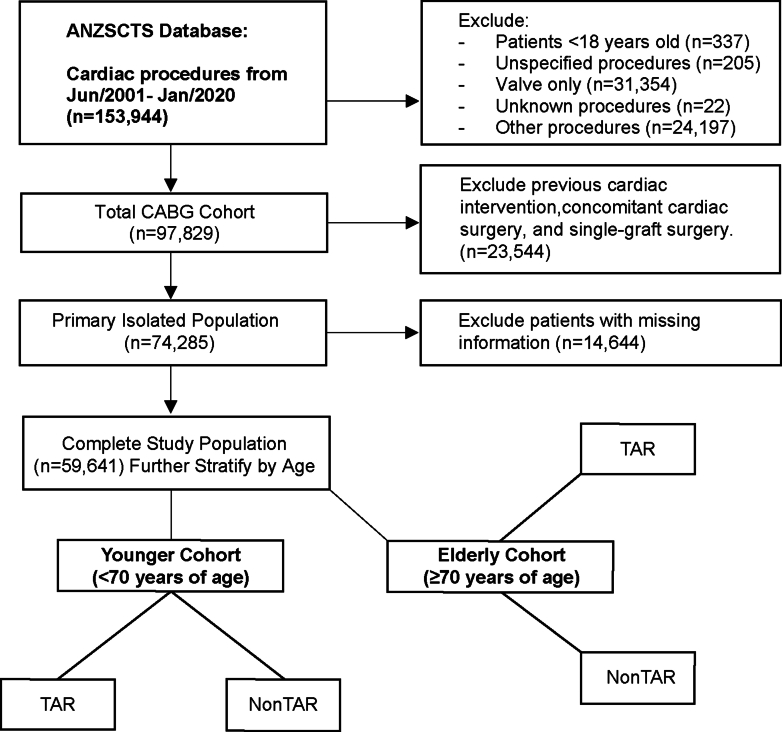
**Patient Selection Process From ANZSCTS Registry** Survival comparison between TAR and non-TAR were conducted across age-stratified patient cohorts. ANZSCTS = the Australian and New Zealand Society of Cardiac and Thoracic Surgeons; CABG = coronary artery bypass grafting; non-TAR = the use of at least 1 saphenous vein graft in CABG operation; TAR = total arterial revascularization.

Of the 153,944 patient records in the ANZSCTS Cardiac Surgery Registry with cardiac procedures performed between 2001 and 2020, a total of 59,641 patients met the eligibility criteria, having undergone primary isolated CABG operations with at least 2 grafts and complete data available for analysis (mean [SD] age, 65.8 [10.2] years; 48,321 men [81.0%]) (Figure 1). The median postoperative follow-up was 5.0 years (IQR: 2.3-8.6), with a maximum follow-up of 17.9 years.

The study cohort included 22,157 elderly patients (aged ≥70 years at the time of surgery) and 37,484 younger patients (aged <70 years). TAR was performed in 17,324 of 59,641 patients (29.0%). Among the 42,317 patients who underwent non-TAR procedures, 18,039 (42.6%) received multiple arterial grafting (non-TAR-MAG) and 24,278 (57.4%) received single arterial grafting (non-TAR-SAG).

TAR was more commonly performed in patients aged <70 years (32.0%) compared to those ≥70 years (24.0%). Younger patients, as defined in the study cohort, had higher rates of smoking and a slightly higher prevalence of diabetes and hypercholesterolemia. In contrast, elderly patients were more multimorbid with increased rates of hypertension, prior cerebrovascular events, peripheral vascular disease, and arrhythmia. Older patients also had higher serum creatinine levels, a greater prevalence of congestive heart failure, and more complex CAD such as triple-vessel and left main disease. The use of perioperative steroid and anticoagulant was higher in elderly patients. More detailed age-stratified baseline characteristics are presented in Table 1. The proportional hazards assumption was not violated for any statistical comparisons in this study (Supplemental Table 1).

**Table 1:** Baseline Characteristics of Patients by Age Group

	Age <70 y (n = 37,484)	Age ≥70 y (n = 22,157)
TAR	12,013 (32.0)	5,311 (24.0)
Age, y	59.8 ± 7.5	76.0 ± 4.2
Body mass index, kg/m^2^	29.6 ± 7.5	28.1 ± 8.3
Smoking history	25,751 (68.7)	12,839 (58.0)
Diabetes	14,050 (37.5)	7,811 (35.3)
Hypercholesterolemia	30,612 (81.7)	17,704 (79.9)
Creatinine, μmol/L	83.7 ± 31.4	102.6 ± 61.1
Dialysis	604 (1.6)	227 (1.0)
Hypertension	28,771 (76.8)	18,687 (84.3)
Cerebrovascular event	2,719 (7.3)	3,123 (14.1)
Peripheral vascular disease	3,131 (8.4)	3,154 (14.2)
Respiratory disease	3,942 (10.5)	3,065 (13.8)
Myocardial infarction	20,077 (53.6)	11,395 (51.4)
Congestive heart failure	4,099 (10.9)	3,161 (14.3)
CCS ≥3	14,457 (38.6)	8,922 (40.3)
NYHA ≥3	5,957 (15.9)	4,559 (20.6)
Cardiogenic shock	395 (1.1)	259 (1.2)
Resuscitation	228 (0.6)	127 (0.6)
Arrhythmia	2,275 (6.1)	2,731 (12.3)
Left main disease	9,164 (24.4)	6,952 (31.4)
Number of diseased territories	3.3 ± 1.0	3.3 ± 0.9
Single-vessel disease	773 (2.1)	300 (1.4)
Double-vessel disease	8,842 (23.6)	4,999 (22.6)
Triple-vessel disease	27,697 (73.9)	16,769 (75.7)
Left ventricular ejection fraction		
>60%	11,930 (31.8)	10,998 (49.6)
46%-60%	6,837 (18.2)	7,050 (31.8)
30%-45%	5,276 (14.1)	3,364 (15.2)
<30%	1,385 (3.7)	745 (3.4)
Perioperative medications		
Inotropes	696 (1.9)	459 (2.1)
Nitroglycerin	2,149 (5.7)	1,410 (6.4)
Anticoagulants	8,196 (21.9)	5,094 (23.0)
Steroids	427 (1.1)	402 (1.8)
Operative details		
Elective	23,593 (62.9)	13,728 (62.0)
Urgent	12,781 (34.1)	7,707 (34.8)
Number of grafts	3.3 ± 1.0	3.3 ± 0.9
On-pump surgery	35,053 (93.5)	20,670 (93.3)
Years of operation	2,011.9 ± 4.6	2,011.7 ± 4.7

Values are n (%) or mean ± SD.

CCS = Canadian Cardiovascular Society classification; TAR = total arterial revascularization.

### Primary analyses

The IPTW risk adjustment successfully reduced all SMDs to below 0.1 (Table 2, Supplemental Figures 1 and 2), indicating adequate covariate balance between groups. Among elderly patients aged ≥70 years, TAR was associated with significantly improved long-term survival compared to non-TAR procedures (HR: 0.87; 95% CI: 0.81 to 0.92; *P* < 0.001). The absolute survival difference at 10 years was 5.6%, with 10-year survival rates of 60.0% (95% CI: 57.9% to 62.2%) in the TAR group and 54.4% (95% CI: 53.1% to 55.7%) in the non-TAR group (Table 3).

**Table 2:** Age-Stratified Baseline Characteristics of Patients Receiving TAR Versus Non-TAR

	Younger Patients (Age <70 y)				Elderly Patients (Age ≥70 y)			
	TAR (n = 12,013)	Non-TAR (n = 25,471)	SMD	ASMD	TAR (n = 5,311)	Non-TAR (n = 16,846)	SMD	ASMD
Male	10,214 (85.0)	21,406 (84.0)	0.03	0	3,729 (70.2)	12,972 (77.0)	0.15	0
Body mass index, kg/m^2^	29.3 ± 6.2	29.7 ± 8.1	0.05	0.01	28.3 ± 7.6	28.1 ± 8.6	0.03	0.02
Smoking history	8,175 (68.1)	17,576 (69.0)	0.02	0.01	3,061 (57.6)	9,778 (58.0)	0.01	0.01
Diabetes	3,805 (31.7)	10,245 (40.2)	0.18	0	1,761 (33.2)	6,050 (35.9)	0.06	0.02
Hypercholesterolemia	9,843 (81.9)	20,769 (81.5)	0.01	0.01	4,253 (80.1)	13,451 (79.9)	0.01	0.01
Creatinine, μmol/L	91.2 ± 64.1	101.0 ± 93.7	0.13	0	90.9 ± 57.7	83.8 ± 48.2	0.08	0.01
Dialysis	52 (0.4)	552 (2.2)	0.15	0.04	32 (0.6)	195 (1.2)	0.06	0.02
Hypertension	8,882 (73.9)	19,889 (78.1)	0.10	0	4,453 (83.8)	14,234 (84.9)	0.02	0.02
Cerebrovascular event	769 (6.4)	1,950 (7.7)	0.05	0.01	751 (14.1)	2,372 (14.1)	0	0.01
Peripheral vascular disease	901 (7.5)	2,230 (8.8)	0.05	0.01	863 (16.3)	2,291 (13.6)	0.07	0.01
Respiratory disease	1,156 (9.6)	2,786 (10.9)	0.04	0	741 (14.0)	2,324 (13.8)	0	0.01
Myocardial infarction	5,830 (48.5)	14,247 (55.9)	0.15	0	2,466 (46.4)	8,929 (53.0)	0.13	0
Congestive heart failure	1,018 (8.5)	3,081 (12.1)	0.12	0.01	716 (13.5)	2,445 (14.5)	0.03	0.01
CCS ≥3	4,938 (41.1)	9,519 (37.4)	0.08	0.01	2,162 (40.7)	6,760 (40.1)	0.01	0.02
NYHA ≥3	1,884 (15.7)	4,073 (16.0)	0.01	0.01	1,146 (21.6)	3,413 (20.3)	0.03	0.01
Cardiogenic shock	54 (0.5)	341 (1.3)	0.09	0.04	31 (0.6)	228 (1.4)	0.08	0.01
Resuscitation	28 (0.2)	200 (0.8)	0.08	0.01	14 (0.3)	113 (0.7)	0.06	0.02
Arrhythmia	600 (5.0)	1,675 (6.6)	0.07	0.01	651 (12.3)	2,080 (12.4)	0	0.01
Left main disease	2,774 (23.0)	6,386 (25.1)	0.05	0	1,526 (28.7)	5,426 (32.2)	0.08	0.01
Number of diseased territories	3.0 ± 0.9	3.4 ± 1.0	0.43	0.01	3.0 ± 0.9	3.4 ± 0.9	0.44	0
Single-vessel disease	536 (4.5)	237 (0.9)	0.22	0	187 (3.5)	113 (0.7)	0.20	0.01
Double-vessel disease	4,185 (34.8)	4,657 (18.3)	0.38	0.01	1,912 (36.0)	3,087 (18.3)	0.41	0.01
Triple-vessel disease	7,248 (60.3)	20,449 (80.3)	0.45	0.01	3,195 (60.2)	13,574 (80.6)	0.46	0
Left ventricular ejection fraction								
>60%	6,758 (56.3)	11,125 (47.6)	0.17	0	2,788 (52.5)	8,210 (48.7)	0.08	0.02
46%-60%	3,676 (30.6)	8,254 (32.4)	0.04	0.01	1,679 (31.6)	5,371 (31.9)	0.01	0.01
30%-45%	1,313 (10.9)	3,963 (15.6)	0.14	0	711 (13.4)	2,573 (15.8)	0.07	0.02
<30%	266 (2.2)	1,119 (4.4)	0.12	0.01	133 (2.5)	612 (3.6)	0.07	0.01
Perioperative medications								
Inotropes	88 (0.7)	608 (2.4)	0.13	0.02	45 (0.9)	414 (2.5)	0.13	0.01
Nitroglycerin	732 (6.1)	1,417 (5.6)	0.02	0.01	255 (4.8)	1,155 (6.9)	0.09	0
Anticoagulants	2,271 (18.9)	5,935 (23.3)	0.11	0.01	958 (18.0)	4,136 (24.6)	0.16	0
Steroids	118 (1.0)	309 (1.2)	0.02	0.04	83 (1.6)	319 (1.9)	0.03	0.01
Operative details								
Elective	7,748 (64.5)	15,845 (62.2)	0.05	0.01	3,505 (66.0)	10,223 (60.7)	0.11	0.01
Urgent	3,984 (33.2)	8,797 (34.5)	0.03	0.01	1,664 (31.3)	6,043 (35.9)	0.10	0
Number of grafts	3.0 ± 0.9	3.4 ± 1.0	0.43	0.02	3.0 ± 0.9	3.4 ± 0.9	0.47	0.04
On-pump surgery	10,618 (88.4)	24,435 (95.9)	0.28	0	4,520 (85.1)	16,150 (95.9)	0.37	0
Year of operation	2,010.4 ± 5.2	2,012.7 ± 4.2	0.48	0.02	2,010.7 ± 5.2	2,012.1 ± 4.5	0.35	0.01

Values are n (%) or mean ± SD. TAR patients received exclusively arterial conduits whereas non-TAR patients received at least 1 saphenous vein graft.

ASMD = adjusted standardized mean difference; SMD = standardized mean difference; other abbreviations as in Table 1.

**Table 3:** Kaplan-Meier Estimated Survival Rates Stratified by Age and Grafting Strategy for Primary and Secondary Analyses

Age <70 y	TAR (95% CI)	Non-TAR (95% CI)
5-y survival	94.1% (93.2%-95.0%)	92.7% (92.4%-93.1%)
10-y survival	84.9% (83.7%-86.1%)	81.2% (80.3%-82.0%)
12-y survival	80.5% (79.1%-81.9%)	74.8% (73.6%-76.0%)
Age ≥70 y		
5-y survival	84.3% (82.9%-85.6%)	81.5% (80.8%-82.2%)
10-y survival	60.0% (57.9%-62.2%)	54.4% (53.1%-55.7%)
12-y survival	49.4% (47.0%-51.8%)	42.7% (41.1%-44.2%)

Age <70 y	TAR (95% CI)	Non-TAR-MAG (95% CI)	Non-TAR-SAG (95% CI)
5-y survival	94.8% (94.2%-95.3%)	93.6% (93.0%-94.2%)	93.0% (92.4%-93.5%)
10-y survival	85.0% (83.9%-86.1%)	83.3% (82.1%-84.5%)	78.8% (77.3%-80.3%)
12-y survival	80.5% (79.2%-81.8%)	76.0% (74.3%-77.7%)	70.7% (68.5%-73.1%)
Age ≥70 y			
5-y survival	85.2% (84.0%-86.5%)	83.0% (81.8%-84.2%)	81.1% (80.1%-82.1%)
10-y survival	61.5% (59.5%-63.6%)	56.0% (54.0%-58.0%)	53.4% (51.6%-55.4%)
12-y survival	51.2% (48.9%-53.7%)	44.4% (42.2%-46.7%)	40.2% (37.8%-42.7%)

TAR patients received exclusively arterial conduits whereas non-TAR patients received at least 1 saphenous vein graft. The top half of the table represents the primary analyses, whereas the bottom half represents the secondary analyses which further stratified non-TAR group into multiple and single arterial grafting.

MAG = multiple arterial grafting; SAG = single arterial grafting; other abbreviations as in Table 1.

**Figure 2:**
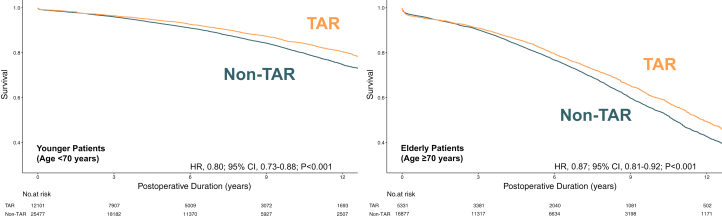
**Adjusted KM Curves Comparing TAR vs Non-TAR in Age-Stratified Populations (Primary Analyses)** Inverse probability of treatment weighting (IPTW) using generalized linear models was applied to achieve covariate balance between groups. Age-stratified survival curves were derived from adjusted models; the numbers at risk represent the IPTW-weighted pseudo-population. The TAR group received exclusively arterial grafts, whereas the non-TAR group received at least 1 saphenous vein graft. KM = Kaplan-Meier; other abbreviations as in Figure 1.

The younger cohort (age <70 years) also had a survival advantage of TAR (HR: 0.80; 95% CI: 0.73-0.88; *P* < 0.001). At 10 years, absolute survival was 84.9% (83.7% to 86.1%) for TAR and 81.2% (80.3% to 82.0%) for non-TAR, representing a 3.7% absolute difference (Table 3). Kaplan-Meier survival curves for both age groups are shown in Figure 2.

### Secondary analyses

**Figure 3:**
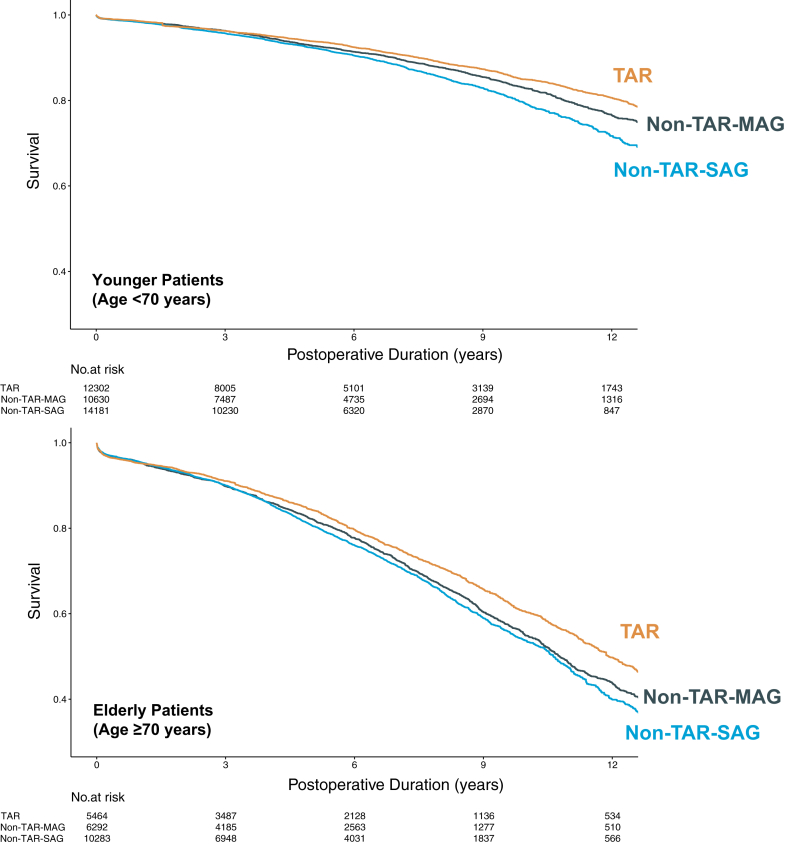
**Adjusted KM Curves Comparing TAR vs Non-TAR-MAG vs Non-TAR-SAG in Age-Stratified Populations (Secondary Analyses)** TAR involves the exclusive use of arterial grafts. Non-TAR-MAG refers to patients receiving ≥2 arterial grafts with at least 1 saphenous vein graft, whereas non-TAR-SAG refers to patients receiving only 1 arterial graft supplemented by saphenous vein grafts. Inverse probability treatment weighting (IPTW) was applied to balance baseline characteristics, and the number at risk reflects the IPTW-adjusted pseudo-population. MAG = multiple arterial grafting (≥2 arterial grafts); SAG = single arterial grafting (1 arterial graft); other abbreviations as in Figure 1 and 2.

Age-stratified analyses comparing TAR, non-TAR-MAG, and non-TAR-SAG revealed a consistent hierarchy in survival outcomes across both younger and older patient groups (Figure 3). Among elderly patients, TAR was associated with significantly lower mortality compared to both non-TAR-MAG (HR: 0.86; 95% CI: 0.80-0.93; *P* < 0.001) and non-TAR-SAG (HR: 0.78; 95% CI: 0.73-0.84; *P* < 0.001). In addition, non-TAR-MAG was associated with a modest but statistically significant reduction in mortality compared to non-TAR-SAG (HR: 0.91; 95% CI: 0.85-0.97; *P* = 0.003).

In the younger cohort, TAR remained associated with superior survival relative to non-TAR-MAG (HR: 0.85; 95% CI: 0.78-0.93; *P* < 0.001) and non-TAR-SAG (HR: 0.68; 95% CI: 0.63-0.75; *P* < 0.001), whereas non-TAR-MAG also demonstrated a significant benefit over non-TAR-SAG (HR: 0.80; 95% CI: 0.74-0.87; *P* < 0.001). These results demonstrate a consistent stepwise reduction in mortality from non-TAR-SAG to non-TAR-MAG to TAR across both age groups (Figure 3, Table 3). All SMDs were reduced below prespecified threshold after IPTW adjustment (Supplemental Figures 3 and 4).

### Sensitivity analyses

Among patients aged ≥70 years, matched analysis of 5,058 pairs showed significantly lower mortality associated with TAR compared to non-TAR procedures (HR: 0.85; 95% CI: 0.84-0.96; *P* = 0.001) (Supplemental Figure 5). In the <70 years cohort, analysis of 11,039 matched pairs in Supplemental Figure 6 similarly demonstrated a significant survival advantage with TAR (HR: 0.77; 95% CI: 0.72-0.83; *P* < 0.001). The matched cohorts are presented in Supplemental Table 2. These findings from PSM analyses were concordant with the main IPTW-adjusted analyses and supported the robustness of the observed treatment effect.

## Discussion

### Findings from study

This binational, registry-based, retrospective cohort study represents the largest age-stratified investigation to date evaluating the long-term survival benefits of TAR in elderly patients (≥70 years) undergoing CABG. Despite longstanding concerns that limited life expectancy may attenuate the benefits of TAR in older adults, our findings demonstrated that TAR provided significant long-term survival benefits in patients aged ≥70 years compared with conventional non-TAR strategies involving SVGs. As expected, a similar survival benefit was observed in the younger cohort (<70 years), reinforcing the established evidence base favoring TAR in this population.

To further elucidate the impact of conduit configuration, our secondary analyses introduced a novel stratification within the non-TAR group, distinguishing between patients who received multiple arterial grafting (non-TAR-MAG) and those who received single arterial grafting (non-TAR-SAG). Across both age groups, we observed a consistent stepwise pattern in survival, with TAR associated with the most favorable outcomes, followed by non-TAR-MAG, and the poorest outcomes seen in non-TAR-SAG (Central Illustration). These findings underscore the dual importance of both maximizing arterial graft use and avoiding SVGs. Importantly, the incremental survival benefit of TAR over non-TAR-MAG highlights that eliminating vein grafts may be more prognostically impactful than simply increasing the number of arterial conduits.

**Central Illustration:**
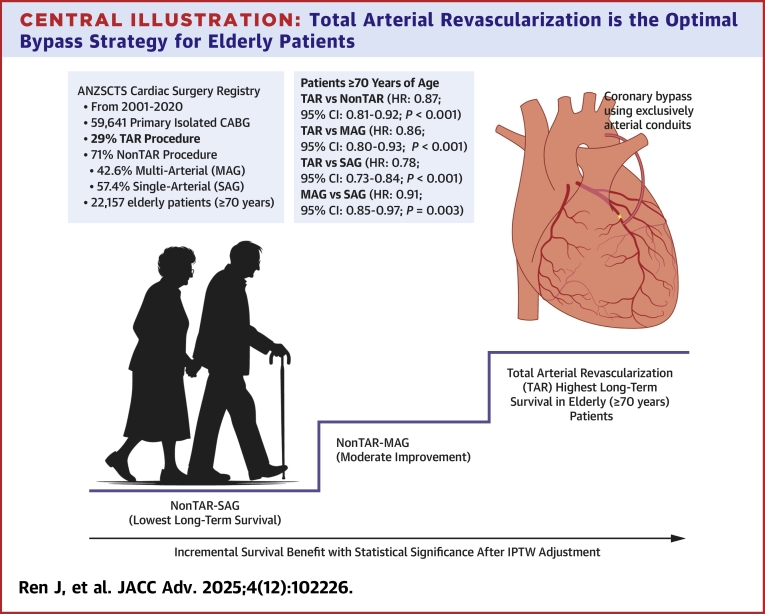
**Total Arterial Revascularization is the Optimal Bypass Strategy for Elderly Patients** Illustration of incremental long-term survival benefit among elderly patients (≥70 years) undergoing different coronary artery bypass grafting strategies, using data from the ANZSCTS Cardiac Surgery Registry. TAR was associated with the highest long-term survival, followed by non-TAR-MAG, and non-TAR-SAG, after IPTW adjustment. ANZSCTS = Australian and New Zealand Society of Cardiac and Thoracic surgeons; CABG = coronary artery bypass grafting; IPTW = inverse probability of treatment weighting; MAG = multiple arterial grafting; SAG = single arterial grafting; TAR = total arterial revascularization.

### Age and bypass configurations

Advanced age is a well-established risk factor for atherosclerosis, often leading to more extensive, calcified, and diffuse CAD in elderly patients. This increased disease burden may deter surgeons from pursuing technically complex arterial-only revascularization, favoring instead conservative strategies that incorporate SVGs. Moreover, several observational studies have questioned the utility of multiarterial grafting in older adults. Kieser et al^13^ reported that the incremental benefit of additional arterial conduits diminishes with age, disappearing beyond 70 years. Similarly, Benedetto et al^14^ found that bilateral internal mammary artery (BIMA) grafting offered a significant survival benefit compared to single internal mammary artery grafting only in patients aged 69 years or younger (HR: 0.49; 95% CI: 0.24-0.98; *P* = 0.04), with no benefit observed in older individuals (HR: 1.27; 95% CI: 0.75-2.14; *P* = 0.37). These findings may have contributed to the ongoing uncertainty regarding the viability of arterial strategies in the elderly.

However, it is important to recognize that prior studies predominantly evaluated BIMA procedures that incorporated supplementary SVGs, which by definition constitute MAG rather than TAR. Although MAG and TAR share the common objective of leveraging the superior patency and durability of arterial conduits, they differ fundamentally in their philosophy. MAG prioritizes increasing the number of arterial grafts, whereas TAR prioritizes complete exclusion of vein grafts (irrespective of the number of arterial graftings). This distinction is central to ongoing debates regarding the optimal revascularization strategy.

Our study offers critical insight into this debate. In addition to confirming the superiority of TAR over SVG-based strategies, we further demonstrated that TAR outperformed MAG in both elderly and younger age groups. Although non-TAR-MAG was associated with improved survival relative to non-TAR-SAG, its persistent inferiority to TAR suggests that the presence of SVGs, regardless of the number of arterial grafts, may attenuate long-term survival benefits. These findings imply that avoidance of vein grafts is a stronger determinant of long-term outcomes than arterial graft count alone. This might help explain why previous BIMA studies failed to detect survival advantages in elderly patients, as the inclusion of SVGs in these cohorts may have masked the true benefit of arterial-only strategies. Post hoc analyses from the ART (Arterial Revascularization Trial)^15^ supported the current findings by demonstrating an incremental benefit across grafting strategies, from SAG to MAG and ultimately to TAR, in terms of reduced 10-year mortality and major adverse cardiac and cerebrovascular events. However, ART did not demonstrate a statistically significant survival benefit of TAR among patients aged >70 years, likely due to insufficient statistical power in this older subgroup. In contrast, our national registry benefits from a substantially higher utilization rate of TAR, approximately 5-fold greater than the international average of 5%,^16^ thereby enhancing the ability to detect treatment effects, even after stratification by age. This increased representation of TAR enabled a more robust comparison and found that TAR conferred superior long-term survival benefits in elderly patients over both SAG and MAG when used with supplementary SVG.

### Study Limitations

This study has several limitations inherent to its retrospective, observational design. Although IPTW and PSM were applied to minimize confounding, the potential for residual confounding due to unmeasured or unrecorded variables cannot be fully excluded. Key factors such as surgeon experience, institutional clustering, intraoperative decision-making, conduit quality, and detailed anatomical information (eg, coronary target vessel quality and size) were not available in the registry data set and may have influenced both conduit selection and long-term outcomes. Although TAR can be achieved without significant alteration to a surgeon’s standard operative technique, by substituting an arterial conduit in place of a venous conduit, routine TAR would require some patients to receive complex arterial graft reconstructions which requires additional surgeon skills and learning curve. In addition, although our adjustment included multiple indicators of clinical status (eg, age, comorbidities, and NYHA class), frailty—which is increasingly recognized as a key predictor of surgical outcomes in elderly populations—was not directly captured, as it is not routinely collected in the ANZSCTS registry or most international surgical data sets. The absence of standardized frailty metrics may limit the granularity of risk adjustment in elderly patients. Furthermore, the registry did not capture postoperative complications or longitudinal clinical events such as graft failure, myocardial infarction, or repeat revascularization, which may have provided further insight into mechanisms underlying observed survival differences. Nevertheless, the use of robust statistical adjustment methods in a large, high-quality, prospectively maintained binational registry provides a strong degree of confidence in the validity and generalizability of the observed associations. Further validation is anticipated from the recently initiated multicenter randomized TA (Total Arterial) trial, designed to compare the angiographic and clinical outcomes of TAR and non-TAR procedures in Australia.^17^

## Conclusions

TAR was consistently associated with improved long-term survival compared to conventional grafting strategies involving vein grafts, including MAG, in both elderly (≥70 years) and younger (<70 years) patients. These findings question prevailing assumptions regarding the limited benefit of arterial grafting in older adults and suggest that the complete avoidance of SVGs may be a key determinant of long-term survival, irrespective of patient age. Although further prospective studies are warranted, these results support broader consideration of total arterial strategies in contemporary surgical practice in selected older patients.

Perspectives **COMPETENCY IN MEDICAL KNOWLEDGE:** This study advances medical knowledge and patient care by demonstrating that TAR significantly improves long-term survival in elderly patients. It supports evidence-based conduit selection to optimize outcomes in an aging surgical population.

**TRANSLATIONAL OUTLOOK:** This study addresses a key evidence gap in elderly surgical patients by demonstrating the long-term survival benefit of TAR. Future research should focus on prospective trials and implementation strategies to increase TAR adoption, particularly in older adults who remain under-represented in surgical innovation.

## Funding support and author disclosures

The ANZSCTS Cardiac Surgery Database Program is funded by the Department of Health (VIC), the 10.13039/501100008878Clinical Excellence Commission (NSW), 10.13039/100010230Queensland Health (QLD), and funding from individual units. ANZSCTS Database Research activities are supported through a National Health and Medical Research Council Principal Research Fellowship (GNT 1136372) and Program Grant (GTN 1092642). The authors have reported that they have no relationships relevant to the contents of this paper to disclose.
