# Supplementary material for: Long-Term Survival Advantage of Total Arterial Revascularization in Elderly Patients Following Coronary Artery Bypass Grafting
Source: JACC Adv. 2025 Oct 8;4(12):102226. doi: 10.1016/j.jacadv.2025.102226 (PMC12805177; doi:10.1016/j.jacadv.2025.102226)
Supplement: Supplemental Material [file mmc1.pdf]

Supplemental Material

Justin Ren, PhD[1,2] Christopher M. Reid, MSc, PhD[3,4] Julian A. Smith MBMS, MSurgEd[5,6] Colin Royse, MBBS, MD[1,7,8] Dion Stub, MBBS, PhD[4,9,10] Wiliam Chan, MBBS, PhD[9,10,11] David M. Kaye, MBBS, PhD[9,10] Jason E Bloom, MBBS, PhD[9,10] Nilesh Srivastav, MBBS, MCh[1,12] Andrea Bowyer, MBBS, PhD[1,7] David H. Tian, MD, PhD[1,13] Lavinia Tran, PhD[4] Jenni Williams-Spence, PhD[4] Doa El-Ansary, PhD[1,14,15] Alistair Royse, MBBS, MD[1,2,15]

1. Department of Surgery, University of Melbourne, Melbourne, Australia
2. Department of Cardiothoracic Surgery, Royal Melbourne Hospital, Melbourne, Australia
3. Population Health, Curtin University, Perth, Australia
4. School of Public Health and Preventive Medicine, Monash University, Melbourne, Australia
5. Department of Surgery, Monash University, Melbourne, Australia
6. Department of Cardiothoracic Surgery, Monash Health, Melbourne, Australia
7. Department of Anesthesia and Pain Management, The Royal Melbourne Hospital, Melbourne, Australia
8. Outcomes research, University of Texas Health, Houston, USA
9. Department of Cardiology, The Alfred Hospital, Melbourne, Australia
10. Baker Heart and Diabetes Institute, Melbourne, Australia
11. Department of Cardiology, Western Health, Melbourne, Australia
12. Heart Vascular and Thoracic Institute, Cleveland Clinic Abu Dhabi, Abu Dhabi, United Arab Emirates
13. Department of Anesthesia and Perioperative Medicine, Westmead Hospital, Sydney, Australia
14. School of Biomedical and Health Sciences, RMIT, Melbourne, Australia
15. Department of Surgery, Universiti Kebangsaan Malaysia, Kuala Lumpur, Malaysia

Address for correspondence
Prof Alistair Royse MBBS, MD, FRACS, FCSANZ, GAICD
Department of Surgery, The University of Melbourne
PO Box 2135, Royal Melbourne Hospital
Carlton, Victoria 3050
Email: Alistair.Royse@gmail.com
Fax: +61386794445
Cell: +61418554135
Twitter: @AlistairRoyse

Supplemental Table 1. Schoenfeld Residuals for Cox Proportional Hazards Model Assumptions Across All Comparisons

Primary Analyses	**P-value**
TAR vs. NonTAR (<70 years old)	0.86
TAR vs. NonTAR (≥70 years old)	0.85
Secondary Analyses	
TAR vs. NonTAR-MAG vs NonTAR-SAG (<70 years old)	0.89
TAR vs. NonTAR-MAG vs NonTAR-SAG (≥70 years old)	0.70
Sensitivity Analyses (propensity score matching)	
TAR vs. NonTAR (<70 years old)	0.99
TAR vs. NonTAR (≥70 years old)	0.92

P-values represent tests of the proportional hazards assumption based on Schoenfeld residuals for each Cox model. A value >0.05 indicates no violation of the assumption. TAR, total arterial revascularization; NonTAR, non–total arterial revascularization; MAG, multiple arterial grafting; SAG, single arterial grafting.

Supplemental Table 2. Age-Stratified Baseline Characteristics of Propensity Score Matched Patients in Sensitivity Analysis

**Characteristics**<br>Patients, No. (%)	**Younger Patients (<70 years old)**			**Elderly Patients (≥70 years old)**		
	**TAR**	**NonTAR**	**SMD**	**TAR**	**NonTAR**	**SMD**
Total sample size	11039	11039	/	5058	5058	/
Male Sex	59.0 ± 7.7	60.1 ± 7.5	0.01	75.9 ± 4.3	76.2 ± 4.2	0.02
Body mass index, mean ± SD	29.4 ± 6.3	29.4 ± 6.8	0	28.3 ± 5.8	28.3 ± 11.6	0.01
Smoking history	7550 (68.4)	545 (4.9)	0	2920 (57.7)	2964 (58.6)	0.02
Diabetes	3633 (32.9)	3665 (33.2)	0.01	1692 (33.5)	1684 (33.3)	0
Hypercholesterolemia	9045 (81.9)	9041 (81.9)	0	4051 (80.5)	4026 (79.6)	0.01
Creatinine, mean ± SD, μmol/L	58.2 ± 41.4	59.6 ± 39.9	0	58.7 ± 38.2	100.2 ± 64.0	0.02
Dialysis	52 (0.5)	63 (0.6)	0.02	31 (0.6)	38 (0.8)	0.02
Hypertension	8241 (74.7)	8276 (75.0)	0.01	4242 (83.9)	4229 (83.6)	0.01
Cerebrovascular event	720 (6.5)	739 (6.7)	0.01	724 (14.3)	705 (13.9)	0.01
Peripheral vascular disease	850 (7.7)	879 (8.0)	0.01	807 (16.0)	852 (16.8)	0.02
Respiratory disease	1066 (9.7)	1074 (9.7)	0	701 (13.9)	716 (14.2)	0.01
Myocardial infarction	5462 (49.5)	5457 (49.4)	0	2367 (46.8)	2355 (46.6)	0
Congestive heart failure	962 (8.7)	979 (8.9)	0.01	683 (13.5)	672 (13.3)	0.01
CCS ≥3	4509 (40.9)	4424 (40.1)	0.02	2054 (40.6)	2081 (41.1)	0.01
NYHA ≥3	1695 (15.4)	1688 (15.3)	0	1086 (21.5)	1115 (22.0)	0.01
Cardiogenic shock	52 (0.5)	59 (0.5)	0.01	30 (0.6)	34 (0.7)	0.01
Resuscitation	28 (0.3)	25 (0.2)	0.01	14 (0.3)	21 (0.4)	0.03
Arrhythmia	561 (5.1)	545 (4.9)	0.01	618 (12.2)	622 (12.3)	0
Left main disease	2253 (19.2)	2639 (23.9)	0.01	1470 (29.0)	1465 (29.0)	0
Number of diseased territories, mean ± SD	3.1 ± 0.9	3.1 ± 0.9	0.03	3.0 ± 0.9	3.0 ± 0.9	0.01
Single-vessel disease	297 (2.7)	224 (2.0)	0.03	127 (2.5)	102 (2.0)	0.03
Double-vessel disease	3580 (32.4)	3529 (32.0)	0.01	1746 (34.5)	1778 (35.2)	0.01
Triple-vessel disease	7119 (64.5)	7241 (65.6)	0.02	3168 (62.6)	3161 (62.5)	0
Left ventricular ejection fraction						
>60%	6119 (55.4)	6104 (55.3)	0	2646 (52.3)	2658 (52.6)	0
46%-60%	3412 (30.9)	3407 (30.9)	0	1564 (30.9)	1574 (31.1)	0.01
30%-45%	1250 (11.3)	1257 (11.4)	0	685 (13.5)	685 (13.5)	0
<30%	258 (2.3)	271 (2.5)	0.01	131 (2.6)	141 (2.8)	0.01
Perioperative medications						
Inotropes	87 (0.8)	94 (0.9)	0.01	45 (0.9)	46 (0.9)	0
Nitroglycerin	638 (5.8)	600 (5.4)	0.01	248 (4.9)	247 (4.9)	0
Anticoagulants	2114 (19.2)	2095 (19.0)	0	930 (18.4)	944 (18.7)	0.01
Steroids	110 (1.0)	115 (1.0)	0	81 (1.6)	81 (1.6)	0
Operative details						
Elective	7094 (64.3)	7205 (65.3)	0.02	3328 (65.8)	3307 (65.4)	0.01
Urgent	3685 (33.4)	3590 (32.5)	0.02	1595 (31.5)	1612 (31.9)	0.01
Number of grafts, mean ± SD	3.1 ± 0.95	3.1 ± 0.88	0.01	3.0 ± 0.9	3.0 ± 0.9	0.01
On-pump surgery	10060 (91.1)	10218 (92.6)	0.04	4446 (87.9)	4510 (89.2)	0.04
Year of operation, mean ± SD	2010.8 ± 5.1	2011.0 ± 4.5	0.04	2010.6 ± 5.1	2010.6 ± 4.7	0.01

TAR patients received exclusively arterial conduits whereas NonTAR patients received at least one saphenous vein graft. TAR, total arterial revascularization; SMD, standardized mean difference after matching; LVEF, left ventricular ejection fraction; CCS, Canadian Cardiovascular Society classification; NYHA, New York Heart Association classification.

Supplemental Figure 1. Love Plots of Standardized Mean Differences Before and After IPTW Adjustment for Patients ≥70 Years of Age (TAR versus NonTAR)

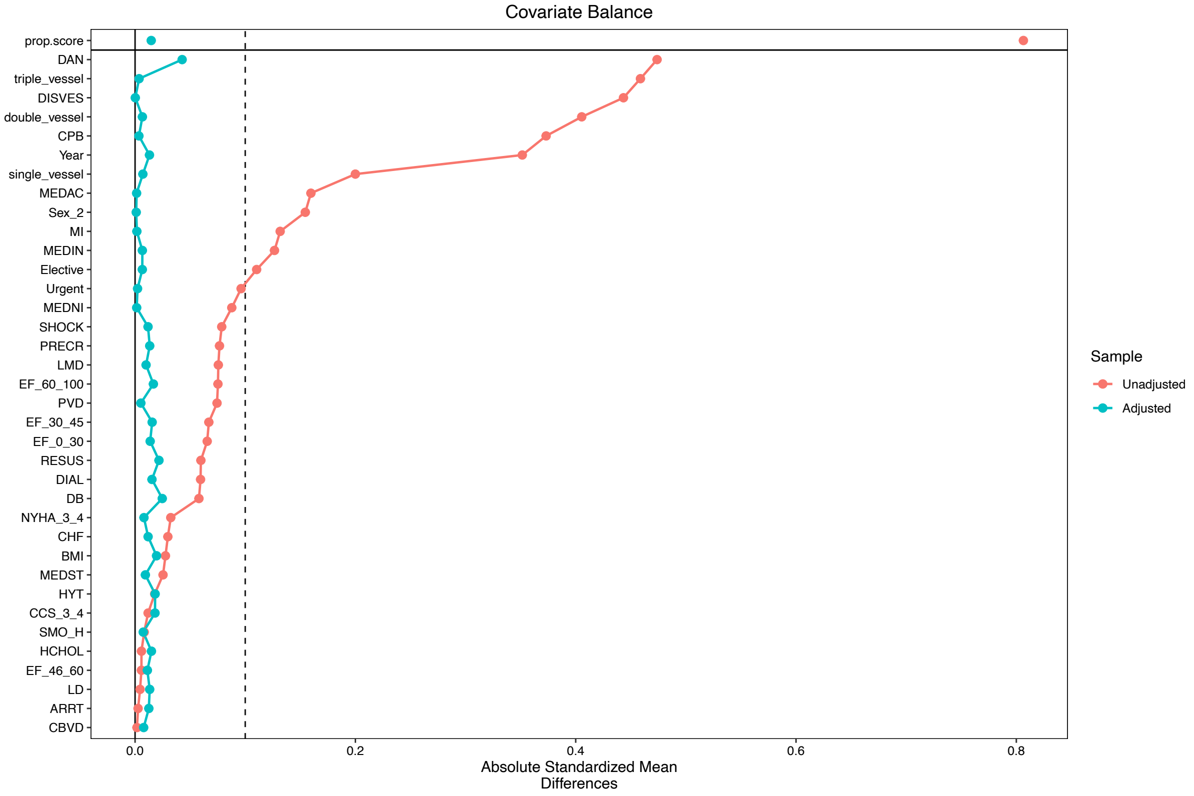

IPTW, inverse probability treatment weighting; GBM, generalized boosted modelling; DAN, number of grafts; triple_vessel, triple vessel disease; DISVES, number of diseased territories; double_vessel, double vessel disease; single_vessel, single vessel disease, CPB, cardiopulmonary bypass; Year, year of operation; AGE, patient age; MEDIN, inotropes; MEDAC, anticoagulation therapy; DB, diabetes mellitus; MI, myocardial infarction; NYHA_3_4, New York Heart Association Classification ≥3; HYT, hypertension; MEDNI, intravenous nitrates; DIAL, dialysis; PRECR, preoperative creatinine level; CCS_3_4, Canadian Cardiovascular Society (CCS) classification ≥3; SMO_H, smoking history; CBVD, cerebrovascular disease; RESUS, resuscitation; LMD, left main disease; MEDST, steroids; ARRT, arrhythmia; CHF, history of congestive heart failure; HCHOL, hypercholesterolaemia; LD, respiratory disease; PVD, peripheral vascular disease; BMI, body mass index.

Supplemental Figure 2. Love Plots of Standardized Mean Differences Before and After IPTW Adjustment for Patients <70 Years of Age (TAR versus NonTAR)

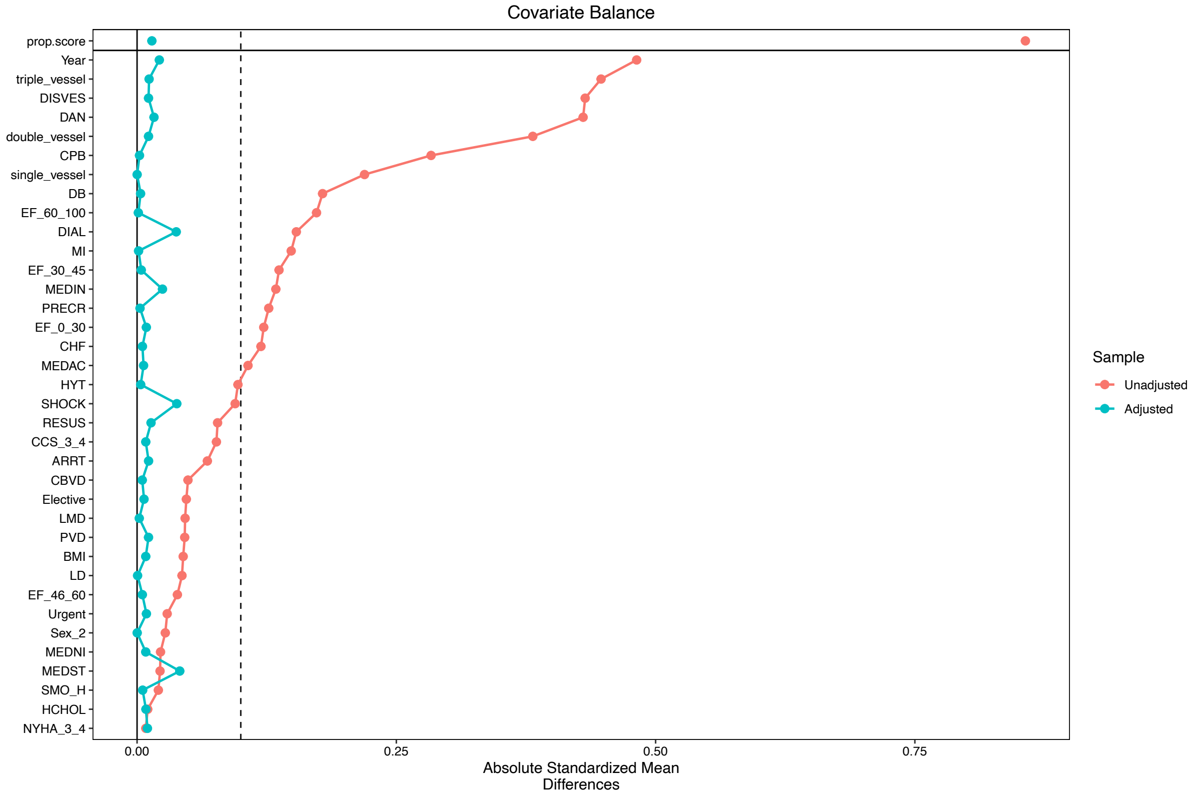

IPTW, inverse probability treatment weighting; GBM, generalized boosted modelling; DAN, number of grafts; triple_vessel, triple vessel disease; DISVES, number of diseased territories; double_vessel, double vessel disease; single_vessel, single vessel disease, CPB, cardiopulmonary bypass; Year, year of operation; AGE, patient age; MEDIN, inotropes; MEDAC, anticoagulation therapy; DB, diabetes mellitus; MI, myocardial infarction; NYHA_3_4, New York Heart Association Classification ≥3; HYT, hypertension; MEDNI, intravenous nitrates; DIAL, dialysis; PRECR, preoperative creatinine level; CCS_3_4, Canadian Cardiovascular Society (CCS) classification ≥3; SMO_H, smoking history; CBVD, cerebrovascular disease; RESUS, resuscitation; LMD, left main disease; MEDST, steroids; ARRT, arrhythmia; CHF, history of congestive heart failure; HCHOL, hypercholesterolaemia; LD, respiratory disease; PVD, peripheral vascular disease; BMI, body mass index.

Supplemental Figure 3. Love Plots of Standardized Mean Differences Before and After IPTW Adjustment for Patients ≥70 Years of Age (TAR versus NonTAR-MAG versus NonTAR-SAG)

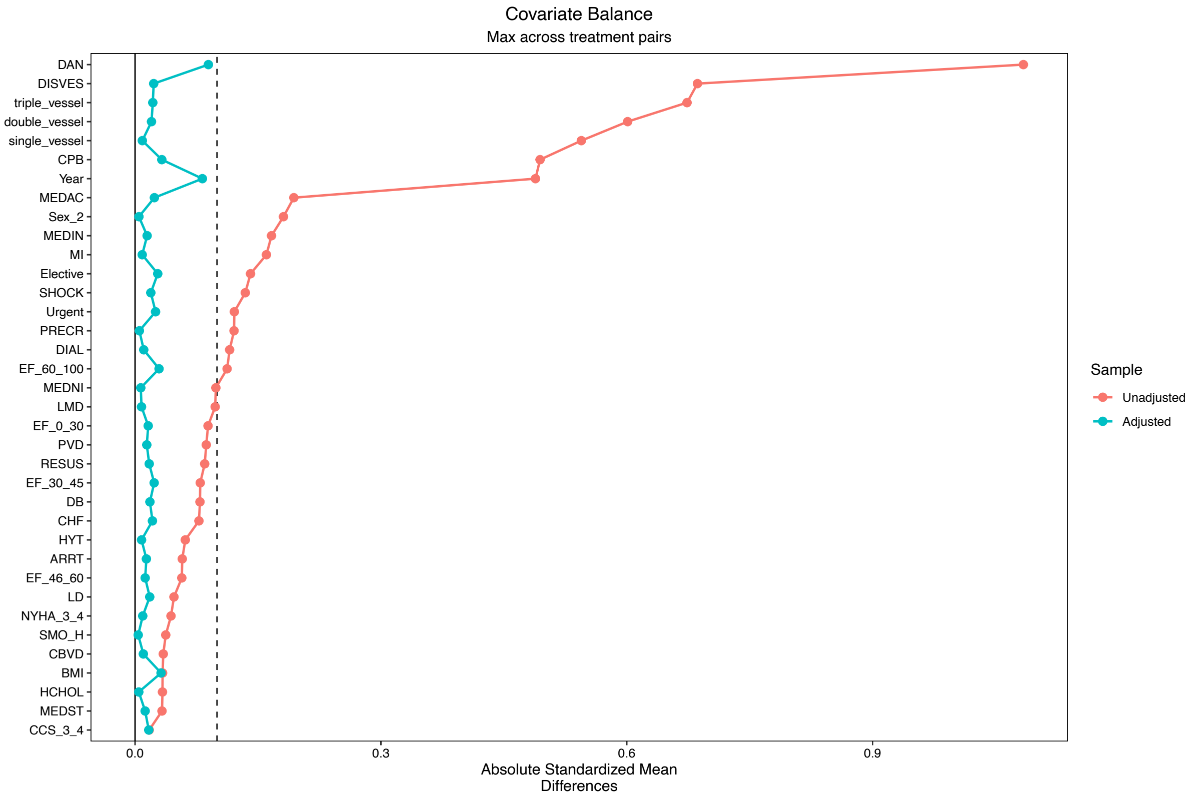

For comparisons involving three groups (TAR, NonTAR-MAG, and NonTAR-SAG), the maximum standardized mean differences across all pairwise contrasts were reported for each covariate. IPTW, inverse probability treatment weighting; GBM, generalized boosted modelling; DAN, number of grafts; triple_vessel, triple vessel disease; DISVES, number of diseased territories; double_vessel, double vessel disease; single_vessel, single vessel disease, CPB, cardiopulmonary bypass; Year, year of operation; AGE, patient age; MEDIN, inotropes; MEDAC, anticoagulation therapy; DB, diabetes mellitus; MI, myocardial infarction; NYHA_3_4, New York Heart Association Classification ≥3; HYT, hypertension; MEDNI, intravenous nitrates; DIAL, dialysis; PRECR, preoperative creatinine level; CCS_3_4, Canadian Cardiovascular Society (CCS) classification ≥3; SMO_H, smoking history; CBVD, cerebrovascular disease; RESUS, resuscitation; LMD, left main disease; MEDST, steroids; ARRT, arrhythmia; CHF, history of congestive heart failure; HCHOL, hypercholesterolaemia; LD, respiratory disease; PVD, peripheral vascular disease; BMI, body mass index.

Supplemental Figure 4. Love Plots of Standardized Mean Differences Before and After IPTW Adjustment for Patients <70 Years of Age (TAR versus NonTAR-MAG versus NonTAR-SAG)

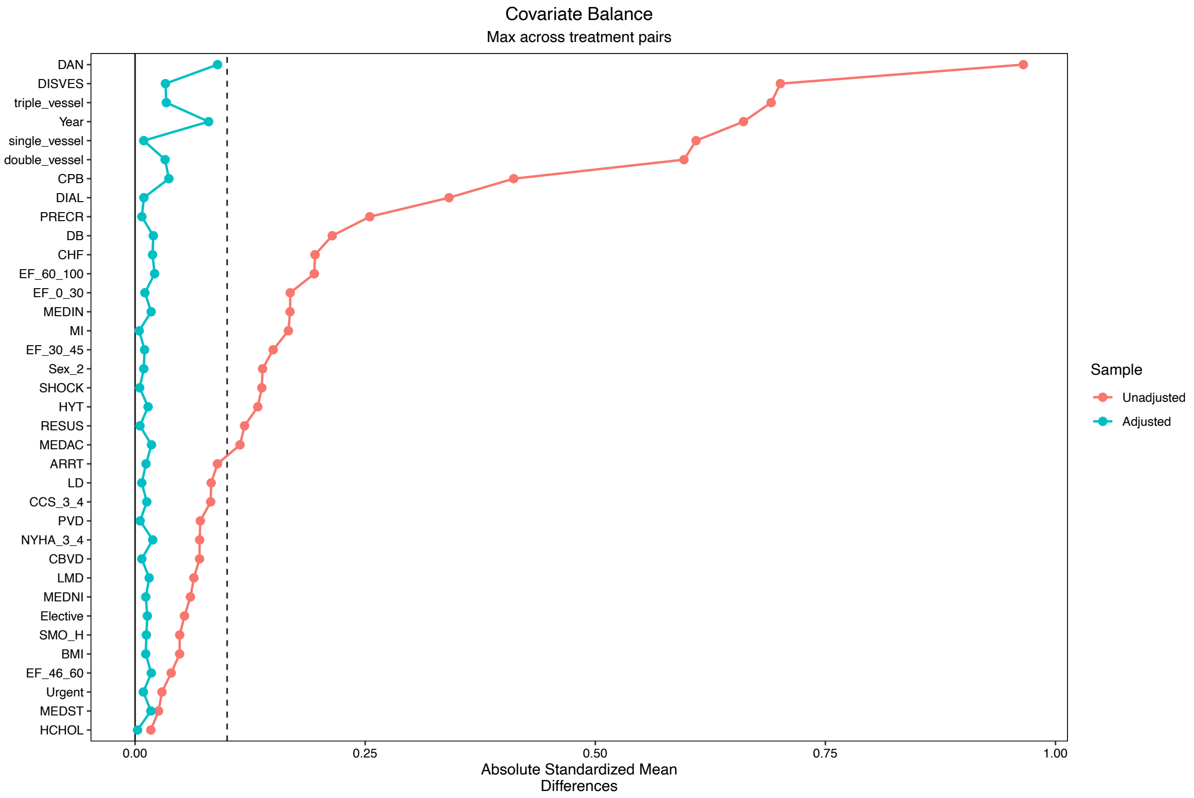

For comparisons involving three groups (TAR, NonTAR-MAG, and NonTAR-SAG), the maximum standardized mean differences across all pairwise contrasts were reported for each covariate. IPTW, inverse probability treatment weighting; GBM, generalized boosted modelling; DAN, number of grafts; triple_vessel, triple vessel disease; DISVES, number of diseased territories; double_vessel, double vessel disease; single_vessel, single vessel disease, CPB, cardiopulmonary bypass; Year, year of operation; AGE, patient age; MEDIN, inotropes; MEDAC, anticoagulation therapy; DB, diabetes mellitus; MI, myocardial infarction; NYHA_3_4, New York Heart Association Classification ≥3; HYT, hypertension; MEDNI, intravenous nitrates; DIAL, dialysis; PRECR, preoperative creatinine level; CCS_3_4, Canadian Cardiovascular Society (CCS) classification ≥3; SMO_H, smoking history; CBVD, cerebrovascular disease; RESUS, resuscitation; LMD, left main disease; MEDST, steroids; ARRT, arrhythmia; CHF, history of congestive heart failure; HCHOL, hypercholesterolaemia; LD, respiratory disease; PVD, peripheral vascular disease; BMI, body mass index.

Supplemental Figure 5. Propensity-Score Matched Kaplan-Meier Survival Curves Comparing TAR versus Non-TAR in Elderly Patients (Sensitivity Analyses)

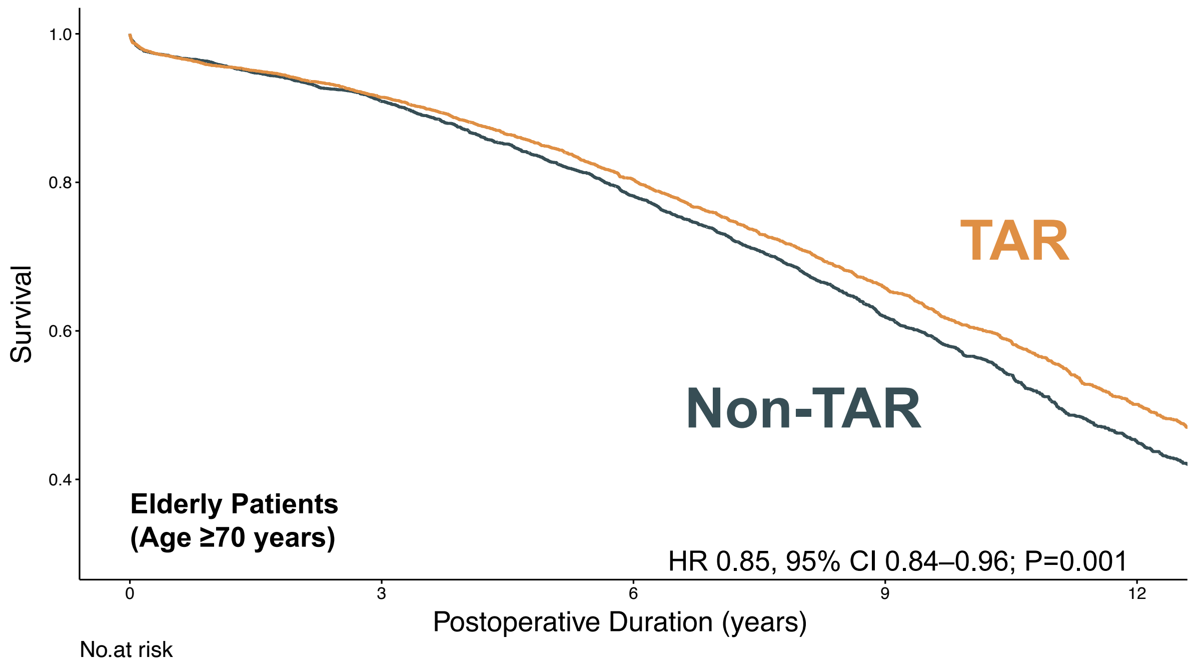

No.at risk

TAR	5058	3624	2377	1346	674
Non-TAR	5058	3764	2422	1279	509

TAR, total arterial revascularization; HR, hazard ratio; CI, confidence interval. The TAR group received exclusively arterial grafts, whereas the non-TAR group received at least 1 saphenous vein graft.

Supplemental Figure 6. Propensity-Score Matched Kaplan-Meier Survival Curves Comparing TAR versus Non-TAR in Younger Patients (Sensitivity Analyses)

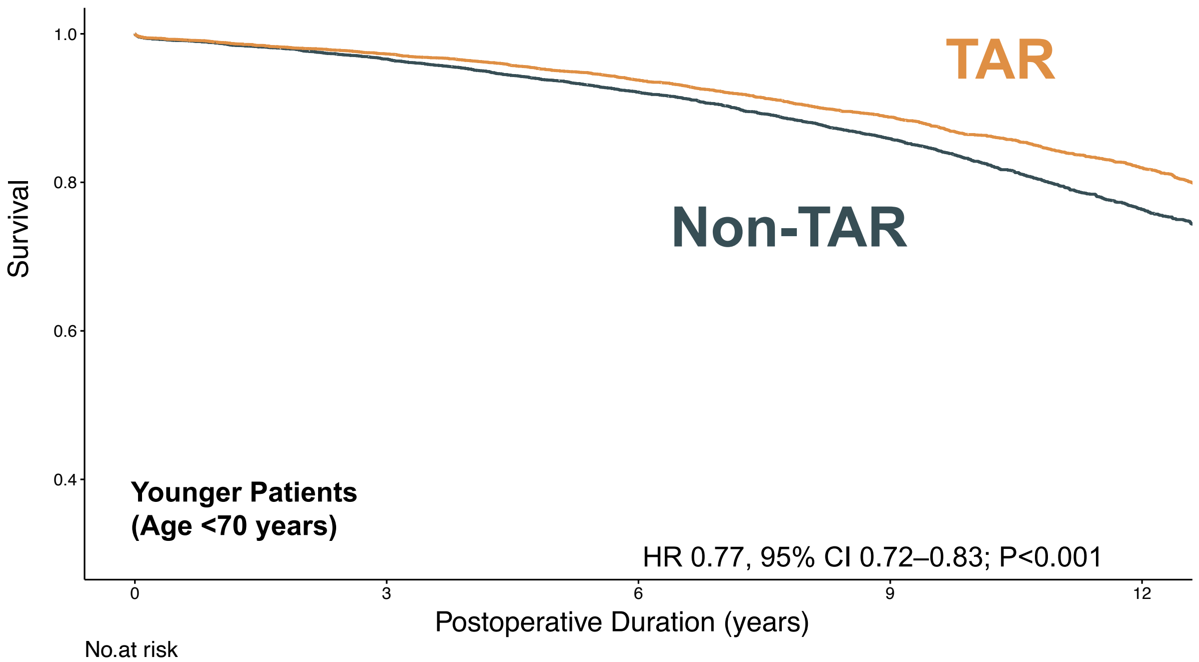

No.at risk

TAR	11039	8300	5827	3839	2198
Non-TAR	11039	8745	5981	3331	1472

TAR, total arterial revascularization; HR, hazard ratio; CI, confidence interval. The TAR group received exclusively arterial grafts, whereas the non-TAR group received at least 1 saphenous vein graft.
